# Green Synthesis of Silver Nanoparticles from *Chlorella vulgaris* Aqueous Extract and Their Effect on *Salmonella enterica* and Chicken Embryo Growth

**DOI:** 10.3390/molecules30071521

**Published:** 2025-03-29

**Authors:** Sebastian Michalec, Wiktoria Nieckarz, Wiktoria Klimek, Agata Lange, Arkadiusz Matuszewski, Klara Piotrowska, Anna Hotowy, Małgorzata Kunowska-Slósarz, Malwina Sosnowska

**Affiliations:** 1 Department of Nanobiotechnology, Institute of Biology, Warsaw University of Life Sciences, 02-787 Warsaw, Poland; s207321@sggw.edu.pl (S.M.); s207323@sggw.edu.pl (W.N.); s200014@sggw.edu.pl (W.K.); agata_lange1@sggw.edu.pl (A.L.); anna_hotowy@sggw.edu.pl (A.H.); 2 Department of Animal Environment Biology, Institute of Animal Sciences, Warsaw University of Life Sciences, 02-787 Warsaw, Poland; arkadiusz_matuszewski@sggw.edu.pl; 3 Department of Animal Breeding and Nutrition, Institute of Animal Sciences, Warsaw University of Life Sciences, 02-787 Warsaw, Poland; klara_piotrowska@sggw.edu.pl (K.P.); malgorzata_kunowska-slosarz@sggw.edu.pl (M.K.-S.)

**Keywords:** algae, antioxidant capacity, bacteria, silver nanoparticles, green synthesis, erythrocytes, chicken embryo

## Abstract

Silver nanoparticles (AgNPs), synthesised using *Chlorella vulgaris* algal extract and silver nitrate, are studied in medicine for their antibacterial properties in poultry. This study assessed the effect of AgNPs on bacterial inhibition and early development and blood parameters in Ross 308 chicken embryos. AgNPs were characterised using transmission electron microscopy, scanning electron microscopy with a focused ion beam, UV–Vis spectroscopy, and a zetasizer. The antibacterial properties of the AgNP colloid against *S. enterica* were assessed using minimal inhibitory concentration, minimal bacterial concentration, and PrestoBlue assays. AgNP colloid (2 mg/L) was injected into egg albumen on day 0. Chicken embryos were incubated for 3 and 16 d. The effect of AgNPs on 3 d old embryos was evaluated based on mortality and somite count using the Hamburger–Hamilton classification. For older embryos, mortality, dimensions, anatomical changes, organ mass, plasma liver enzymes and antioxidants, and red blood cell morphology were determined. Blood samples from the control group embryos were assessed for the impact of AgNPs on hemolysis. AgNPs inhibited *S. enterica* growth at concentrations >6.75 mg/L. A 3 d exposure to AgNPs caused an insignificant decrease in the number of somites without affecting embryo mortality. However, a 16 d exposure to AgNPs reduced live embryos and plasma antioxidants, changed the levels of ALT, AST, and GGT, altered red blood cell morphology, and caused hemolysis. Toxicity of AgNPs was model-dependent, whereby the chicken embryo was more sensitive to AgNPs than the bacterium.

## 1. Introduction

The poultry industry is a significant subsector of animal production, with profound global economic importance [1]. In 2023, the per capita annual consumption of poultry in the United States was estimated to total 52.6 kg, with chicken accounting for 45.9 kg and turkey contributing 6.7 kg [2]. Poultry plays a crucial role in addressing nutritional deficits by providing an economical source of protein [1]. However, feed costs constitute a substantial portion of overall production expenses, ranging from 60 to 70%. Therefore, the search for more economical feed alternatives is becoming increasingly attractive. Moreover, due to restrictions on antibiotics and growth-promoting agents, there is an urgent need to find natural alternatives with antimicrobial properties that do not compromise meat quality or poultry health [3].

*Salmonella* is a genus of Gram-negative rod bacteria belonging to the family Enterobacteriaceae. The bacteria die only at 70 °C. Birds infected by *S. enterica* may be symptomatic or asymptomatic. The lack of clinical signs is associated with the spread of bacteria during evisceration and sale of contaminated poultry carcasses. *S. enterica* causes salmonellosis in humans through the consumption of poultry meat or eggs contaminated with chicken faeces [4]. Therefore, food preparation, poultry farming, and transport and processing of meat and eggs should minimise the spread of *Salmonella*. Salmonellosis is associated with a severe clinical course with gastrointestinal disturbances when transmitted to humans, particularly the *S. enterica* serotype Enteritidis. The irrational use of antibiotics, such as chloramphenicol, tetracycline, streptomycin, sulphonamide, and ampicillin, as growth promoters enhances the acquisition of resistance in some serotypes [5]. Silver [6], iron oxide [7], zinc oxide [8], and gold [9] nanoparticles are a new alternative to widely used antibiotics in the control of different serotypes of *S. enterica* with simultaneous growth of *Lactobacillus* bacteria in the intestine. Thus, nanoparticles have great potential as biocidal agents in cages and housing for chickens [10]. However, little is known whether they can be incorporated into the digestive and reproductive systems of chickens without affecting weight gain and overall health.

Silver has been valued for its antimicrobial properties for centuries and was used in storing water and treating wounds, burns (silver sulphadiazine), ulcers, and eye infections in newborns (silver nitrate) [11]. Minimising silver to a nanometre size (1–100 nm) increases its active surface and enhances its bacteria-penetrating properties. The mechanism of silver nanoparticles (AgNPs) is based on anchoring to the bacterial cell wall, enhancing the release of reactive oxygen, membrane disruption, interaction with the respiratory enzymes and phosphate groups of DNA and proteins, interaction with cellular components, and cell destruction and leakage of cell contents or regulation of key gene expression [10,11,12,13]. AgNPs, whether obtained through conventional methods [14] or green synthesis [15], exhibit antibacterial activity against, for example, methicillin-resistant *Staphylococcus aureus*, with the added benefit of limiting antibiotic resistance.

AgNPs can be synthesised by physical, chemical, and biological methods (green synthesis). In each of these methods, nanoparticles are formed by the reduction of silver ions from salt and subsequent self-assembly of atoms [16]. In green synthesis, the reduction of silver ions takes place by organisms such as plants, algae, bacteria, and fungi [17]. This eco-friendly approach leverages the natural reducing and stabilising agents of organisms, such as amino acids, polyphenols, flavonoids, polysaccharides, terpenoids, alkaloids, and others [17]. Green synthesis methods reduce the use of toxic chemicals, utilise renewable energy sources, and are environmentally friendly. Nanoparticles created by green synthesis, even without sufficient purification, have low toxicity in the environment due to the fact that they contain only substances of natural origin and not toxic reducing agents such as hydrazine [18]. However, the benefits of employing green synthesis extend beyond environmental considerations. Metal nanoparticles produced in this way exhibit much higher biocompatibility compared to physical and chemical synthesis methods. Additionally, extracts from living organisms impart enhanced antibacterial, antifungal, drug delivery, and photodegradation properties to nanoparticles [18,19,20].

*Chlorella vulgaris* is a microalga containing a range of bioactive substances, such as polysaccharides (starch and cellulose), vitamins, proteins, lipids, antioxidants (polyphenols and tocopherols), and pigments such as carotenoids (carotene and xanthophyll), chlorophylls and phycobilins, phycocyanin, and phycoerythrin, used in the synthesis of AgNPs biotically (intracellular) and abiotically (extracellular) [21,22]. Half of the dry mass of algae is protein. Metal salts are reduced inside algal cells by nitrate reductase by nicotinamide adenine dinucleotide + hydrogen- and nicotinamide adenine dinucleotide phosphate-dependent enzymes that are involved in metabolic processes, including photosynthesis, nitrogen fixation, respiration, and pigmentation such as in chlorophyll [22]. The main benefit of using algal extracts lies in their high content of strongly stabilizing polysaccharides, absence of potentially toxic compounds, and presence of diverse compounds such as unsaturated fatty acids and peptides, giving algal extracts a favourable nutritional profile [23]. By utilising algal biomass in the synthesis process, nanoparticles are enriched with unique properties through surface modifications using various functional groups and biomolecules. Unfortunately, not all species of algae allow for the synthesis of metal nanoparticles, and successful synthesis often results in nanoparticles with low size homogeneity [23,24].

AgNPs contribute to enhancing myogenic differentiation and reducing oxidative stress in muscles [25,26]. However, the incorporation of AgNPs into poultry diets must be carefully managed to avoid potential toxicity. In vivo studies conducted on chicken embryos and quail have demonstrated that AgNPs do not significantly impact growth or development nor induce oxidative DNA damage in chicken embryos [27]. However, Zhang et al. [28] observed an increase in embryonic mortality following exposure to AgNPs at a concentration of 3 mg/L. Additionally, prolonged exposure to silver has been shown to reduce plasma antioxidant potential, possibly due to the slow oxidation of nanoparticles and the release of highly reactive silver ions [29]. A study by Ognik et al. [30] similarly reported a marked reduction in blood antioxidant capacity when AgNPs were administered orally to post-hatch chickens. Comparable findings have been observed in other species, including the European rabbit, *Oryctolagus cuniculus* [31], and the Norwegian rat, *Rattus norvegicus* [32]. These findings highlight an urgent need for comprehensive data on the biodistribution and accumulation of AgNPs across diverse research models, considering the varied physicochemical properties of these nanoparticles. A slight change in the dimensions of AgNPs modifies their toxicity, so assessing the toxicity after nanoparticle synthesis is crucial for their commercial application.

Currently, limited research exists on the application of AgNPs in the poultry industry. Thus, the aim of our study was to evaluate the effects of green-synthesised AgNPs on *S. enterica* and on the developmental and blood parameters of Ross 308 chicken embryos.

## 2. Results

### 2.1. Green Synthesis Method Allowed Obtaining Spherical, Stable AgNPs with Negative Charge and High Purity

**Figure 1:**
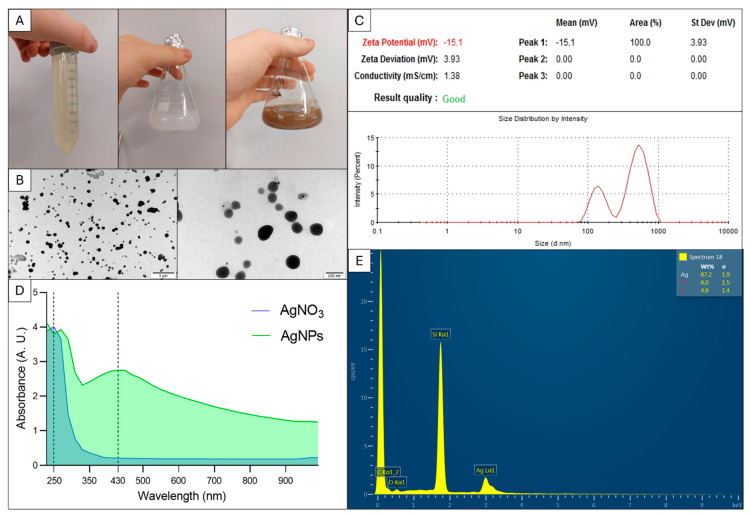
Characterisation of silver nanoparticles (AgNPs) obtained using silver nitrate (AgNO_3_) and *Chlorella vulgaris*: (**A**) Colour change after 3 h of incubation of silver nitrate and algae extract in darkness. (**B**) Morphology of nanoparticles observed under a transmission electron microscope. Scale bar = 200 nm (right side) and 1 µm (left side). (**C**) Colloid stability (table) and nanoparticle size (graph) measured with a zetasizer. (**D**) Ultraviolet−visible spectrum of the AgNPs (green line) and AgNO_3_ (blue line). Dashed vertical lines were added along the *x*-axis to indicate the wavelengths corresponding to the peaks of each spectrum, 250 nm for AgNO_3_ and 430 nm for AgNPs. (**E**) Chemical composition of AgNPs measured by scanning electron microscopy with a focused ion beam.

During the 3 h incubation, the mixture consisting of algae extract and silver nitrate salts changed its colour visibly from green to brown (Figure 1A). It was the first evidence of successful and efficient synthesis of nanoparticles. Transmission electron microscopy images confirmed the presence of spherical AgNPs with diameters ranging from 18 nm to over 200 nm (Figure 1B). The polydispersity index of AgNPs was 0.59 ± 0.13. The AgNP colloid showed a zeta potential of −15.0 ± 0.1 mV, thus medium stability and a negative charge of particles. Results from dynamic light scattering showed the presence of small nanoparticles about 90 nm in size. However, the average hydrodynamic diameter was 412 ± 121 nm (Figure 1C). The UV−Vis spectrum showed a clear peak around 430 nm, which is characteristic of AgNPs but not observed for silver nitrate (Figure 1D). The mass concentration of silver in the AgNP colloid was 87.2%. The colloid also contained residual amounts of other elements such as oxygen, carbon, and silicon (Figure 1E).

### 2.2. Silver Nanoparticles Have Antibacterial Activity Against S. enterica

**Figure 2:**
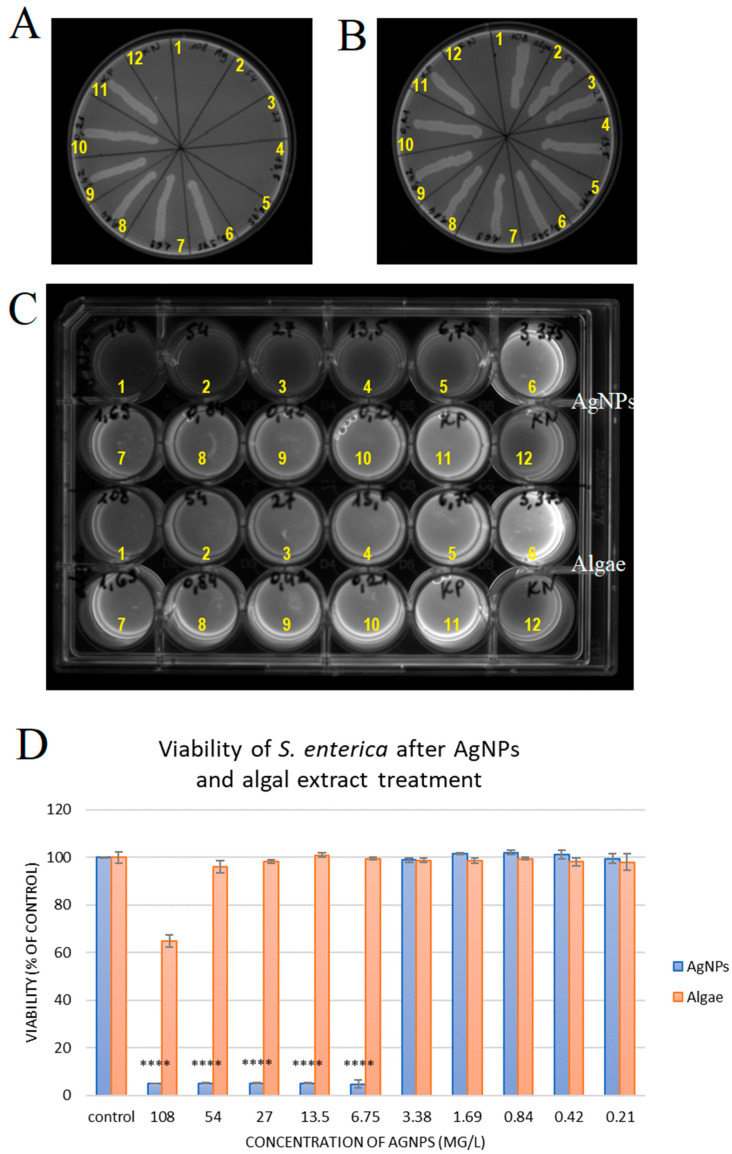
(**A**) Minimum bactericidal concentrations of silver nanoparticles (AgNPs) after 24 h incubation. (**B**) Minimum bactericidal concentrations of algal extract after 24 h incubation. (**C**) Minimum inhibitory concentration and turbidity for different concentrations of AgNPs and algal extract after 24 h incubation. (**D**) PrestoBlue test results after 24 h of incubation of *S. enterica* with AgNPs and algal extract. **** = significant difference (*p* ≤ 0.0001). 1–10: Nanoparticles/algae extract concentrations; 1: 108 mg/L, 2: 54 mg/L, 3: 27 mg/L, 4: 13.5 mg/L, 5: 6.75 mg/L, 6: 3.38 mg/L, 7: 1.69 mg/L, 8: 0.84 mg/L, 9: 0.42 mg/L, 10: 0.21 mg/L, 11: positive control, 12: negative control.

Minimal bactericidal concentration (MBC) and minimal inhibitory concentration (MIC) tests were used to assess the antimicrobial activity of the AgNPs against *S. enterica*. The MBC value for *Salmonella* exposed to different concentrations of nanoparticles (from 108 to 0.21 mg/L) was 6.75 mg/L (Figure 2A). The algal extract corresponding to the concentration of the extract in nanoparticles was not bactericidal (Figure 2B; Table 1). After 24 h of incubation, turbidity was noticed in the test tubes with AgNP concentrations of 3.38, 1.69, 0.84, 0.42, and 0.21 mg/L, indicating bacterial growth. However, at the AgNP concentrations of 6.75, 13.50, 27.00, and 108.00 mg/L, no turbidity was seen, thus exhibiting inhibition of *S. enterica* growth. Sample turbidity following addition of algal extract was observed at all concentrations except the highest (Figure 2C, Table 1).

The percentage viability of *S. enterica* bacteria after incubation with nanoparticles and algal extract is presented in Figure 2D. The PrestoBlue results showed that AgNPs at the five highest concentrations, i.e., 6.75, 13.50, 27.00, 54.00, and 108.00 mg/L, caused a statistically significant (*p* ≤ 0.0001) decrease of about 95% in the viability of *S. enterica*. Lower concentrations of AgNPs did not change the viability of cells compared to the control. The algal extract reduced the viability of bacteria only at the highest concentration by about 35 ± 2%. These results confirm that the created nanoparticles are effective against *S. enterica* at concentrations above 6.75 mg/L.

### 2.3. AgNPs Did Not Change Developmental Stage of Chicken Embryos After 72 H of Incubation

**Figure 3:**
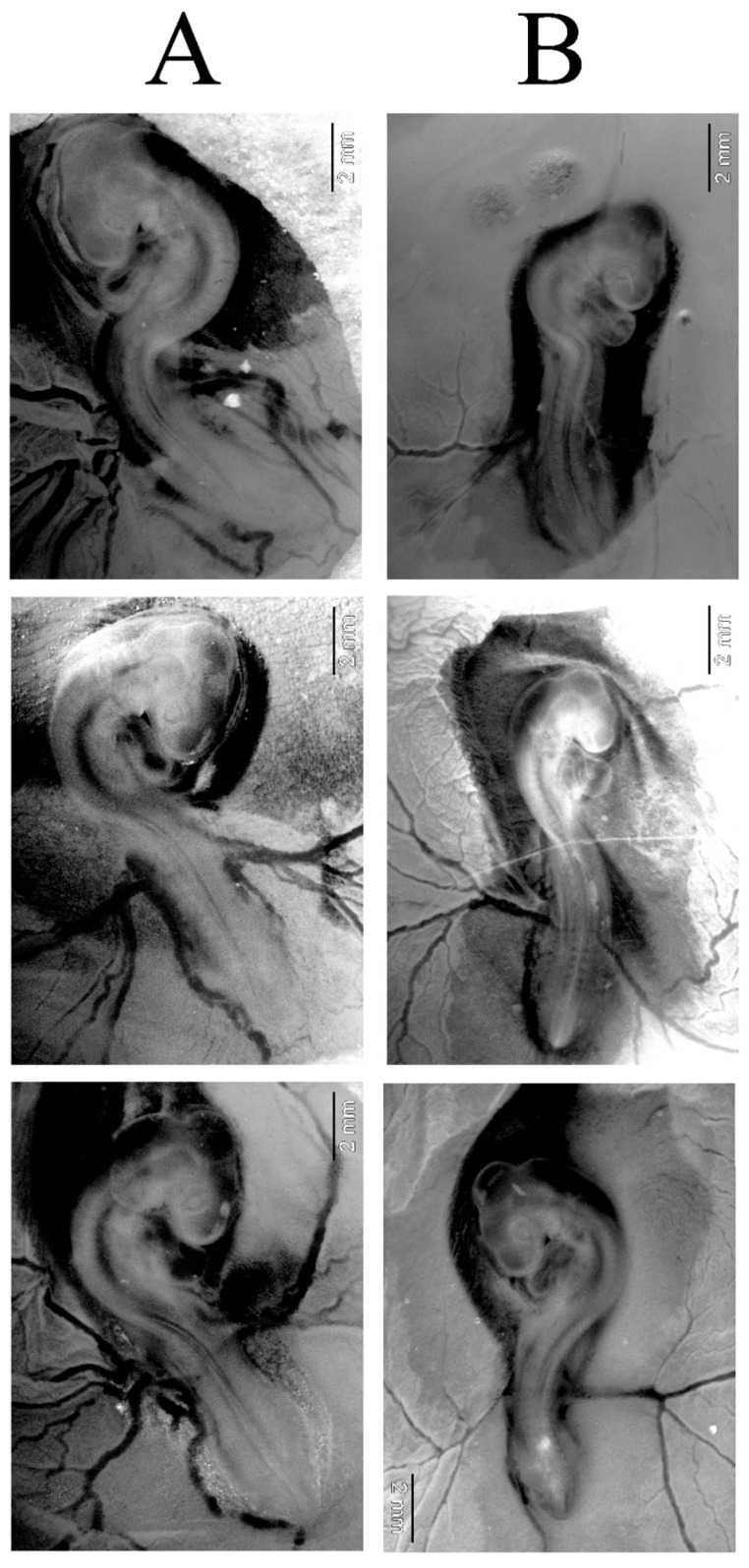
Stereoscopic images of 3 d old chicken embryos after injection with (**A**) phosphate-buffered saline and (**B**) silver nanoparticles at a concentration of 2 mg/L. Photographs were modified with ImageJ version 1.54d and GIMP 2.10.22 software. Scale bar = 2 mm.

Exposure to AgNPs at 2 mg/L was non-toxic to chicken embryos during the early stages of embryogenesis. All embryos in the control and AgNP groups were alive. However, in the AgNP group, the embryos appeared smaller. The count of somite number under a stereoscope revealed approximately 35 somites in the control group compared to about 33 somites in the AgNP group. Thus, embryos showed a slight developmental delay in the AgNP group (Figure 3). However, the number of somites corresponded to developmental stage 18 (approximately 65–69 h) in both groups, according to Hamburger and Hamilton [33]. Furthermore, AgNPs appeared to reduce the vascularisation of the chorioallantoic membrane, which could potentially have an influence on embryo nutrition.

### 2.4. Long Exposure to AgNPs Caused Mortality of Chicken Embryos and Changed Weight of Key Organs

The 16 d old chicken embryos exposed to AgNPs experienced a high mortality rate of 40% (two dead and three alive). In the control group, all embryos were alive. Differences between the control group and the AgNP group varied among body parts (Table 2). The weight of live embryos in the AgNP-treated group did not differ statistically (*p* > 0.05) from the weight of live embryos in the control group. Long-term exposure of embryos to AgNPs resulted in a trend towards greater heart weight compared to the control group, but not significantly (*p* > 0.05). Brain weight was significantly greater (*p* < 0.05) in the control group. Liver weight was significantly greater (*p* < 0.05) in the AgNP group.

The overall appearance of the organs did not differ between the groups, although significant greenish discolouration of the liver and chest deformation were observed in one of the embryos from the AgNP group. This could have been caused by damage to the gallbladder and leakage from it onto the liver lobes. The high mortality rate suggests that prolonged exposure to AgNPs can have more pronounced toxic effects, potentially due to the accumulation of nanoparticles in developing tissues and organs.

### 2.5. AgNPs Changed Liver Enzyme Levels

**Figure 4:**
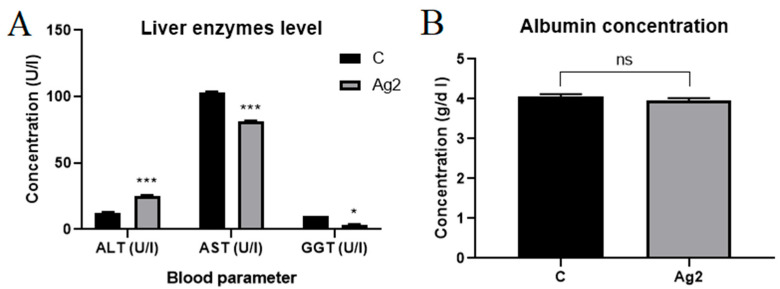
(**A**) Concentration of alanine aminotransferase (ALT), aspartate aminotransferase (AST), and gamma-glutamyltransferase (GGT) in the plasma of 16 d old chicken embryos from the control group (C) compared to the group injected with silver nanoparticles (AgNPs) at a concentration of 2 mg/L, measured using Fuji Dri-Chem NX500 analyser. (**B**) Albumin concentration in the plasma of 16 d old chicken embryos from the control group (C) and the group injected with AgNPs at a concentration of 2 mg/L. * = significantly different (*p* ≤ 0.05); *** = significantly different (*p* ≤ 0.001); ns = not significantly different (*p* > 0.05).

In the next step, we wanted to check whether changes in liver weight were associated with liver enzymes and albumin levels. AgNPs changed liver enzyme parameters and did not change albumin levels in the blood plasma of 16-day-old chicken embryos (Figure 4). Alanine aminotransferase (ALT) levels increased in the AgNP group (25.33 ± 0.47 U/L) compared to the control group (12.67 ± 0.47 U/L). However, the concentration of aspartate aminotransferase (AST) and gamma-glutamyltransferase (GGT) decreased significantly in the study group.

### 2.6. AgNPs Decreased Antioxidant Levels in Chicken Embryo Plasma

**Figure 5:**
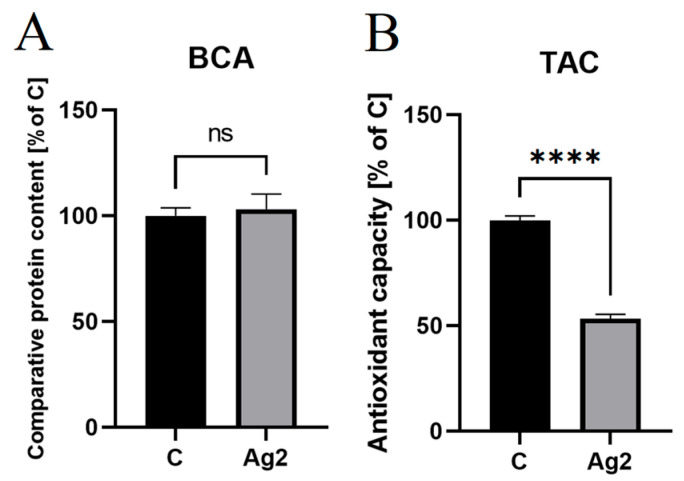
(**A**) Protein content in the plasma of 16 d old chicken embryos from the control group (C) compared to the group injected with silver nanoparticles (AgNPs) at a concentration of 2 mg/L, measured by the bicinchoninic acid (BCA) method. (**B**) Total antioxidant capacity (TAC) in the plasma of 16 d old chicken embryos from the control group and the group injected with AgNPs at a concentration of 2 mg/L. **** = significantly different (*p* ≤ 0.0001); ns = not significantly different (*p* > 0.05).

The results of the bicinchoninic acid assay indicated that the plasma samples did not differ significantly (*p* > 0.05) in total protein content between the experimental groups (Figure 5A). Therefore, the initial samples were used for the experiment, and the protein equalisation step was omitted. The total antioxidant capacity test showed a statistically significant decrease (*p* ≤ 0.0001) in the antioxidant content in the plasma of the AgNP group (53.35 ± 2.06%) compared to the control group (100.00 ± 2.11%) (Figure 5B).

### 2.7. AgNPs Changed Red Blood Cell Morphology

**Figure 6:**
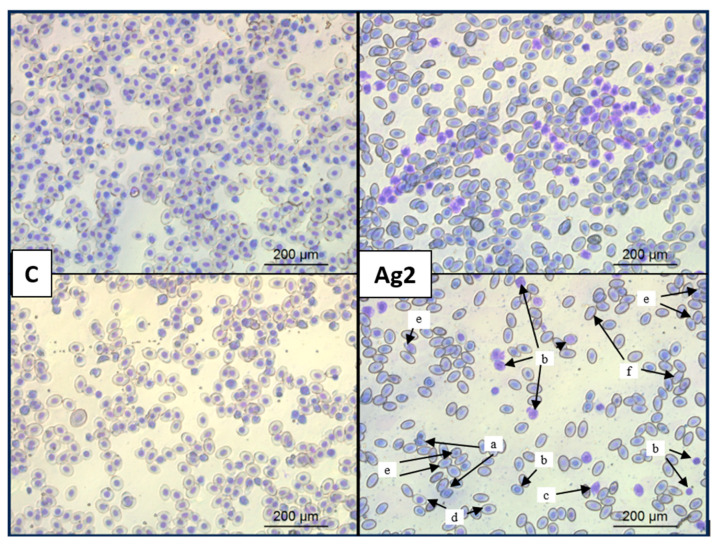
Blood smears under light inverted microscopy from the control group (C) and the silver nanoparticle (AgNP)-treated group after staining with May–Grünwald–Giemsa. Arrows indicate examples of morphological changes in erythrocytes: (a) deformation; (b) polychromatophilic/immature erythrocyte; (c) polychromatophilic poikilocyte; (d) erythrocyte swelling; (e) mitotic erythrocyte; (f) loss of distinct nuclear structures in the erythrocyte. Scale bar = 200 µm.

The evaluation of red blood cells using light microscopy revealed that AgNPs have a detrimental effect on their morphology. Cell membranes were damaged, cell shape became deformed, and the cells lost their characteristic biconcave, elliptical shape. Additionally, AgNPs caused hemagglutination, i.e., the clumping of erythrocytes into larger aggregates. Moreover, exposure to AgNPs induced anisocytosis with an increased presence of polychromatic erythrocytes (immature erythrocytes) compared to the control group, loss of distinct nuclear structures, deformed erythrocytes, and an increased number of mitotic erythrocytes. Selected changes in erythrocyte morphology due to AgNPs are shown in Figure 6.

### 2.8. AgNPs Did Not Cause Lysis of Red Blood Cells

**Figure 7:**
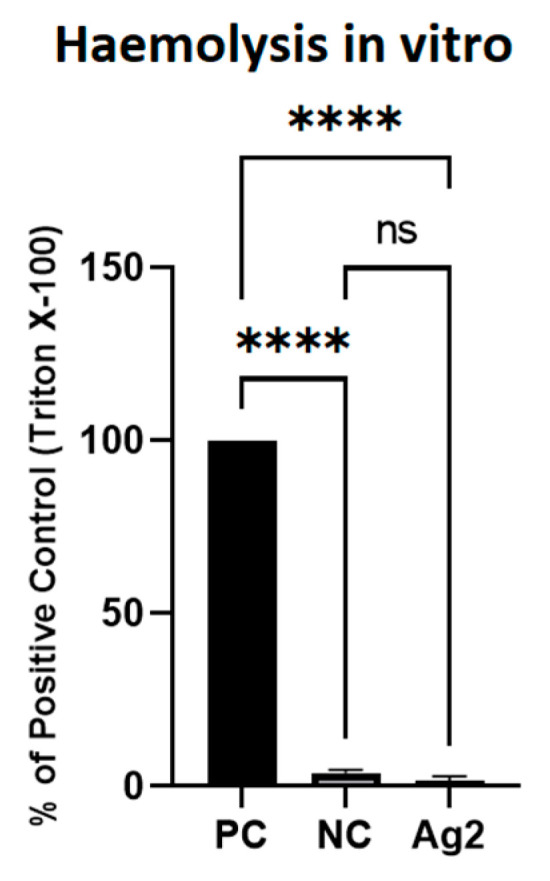
Haemolysis of erythrocytes after 1 h of exposure to Triton X-100 (positive control—PC), phosphate-buffered saline (negative control—NC), and silver nanoparticles (AgNPs) at a concentration of 2 mg/L. **** = significantly different (*p* ≤ 0.0001); ns = not significantly different (*p* > 0.05).

Figure 7 presents the results of the haemolysis test of erythrocytes obtained from the whole blood of 16 d old chicken embryos in the positive control, negative control, and AgNP-treated groups. Erythrocytes exposed for 90 min to AgNPs at 2 mg/L were not significant different (1.49 ± 1.31% of positive control group) from erythrocytes in the negative control group (3.84 ± 0.85% of positive control group). Complete haemolysis was recorded after Triton X-100 was added to the erythrocytes. Thus, AgNPs did not cause erythrocyte lysis after short-term exposure, as observed by the absence of haemoglobin release from the cells.

## 3. Discussion

Our study included three sets of experiments. The first set concerned the synthesis and characterisation of AgNPs produced by using algal extract. The second set was devoted to the antibacterial properties of the resulting nanoparticles. The third set concerned the evaluation of the potential toxicity of AgNPs in a chicken embryo model. These preliminary investigations indicate the possible use of AgNPs in disinfecting facilities intended for breeding chickens for control of *S. enterica*.

The first characteristic feature of efficient synthesis of AgNPs is the change in the colour of the colloid. The synthesised nanoparticles with specific shapes and dimensions characteristically reflect visible light, a phenomenon called surface plasmon resonance [34]. A gradual colour change from light green to brown occurred after mixing 5 mL of *C. vulgaris* extract with 45 mL of 1 mM silver nitrate. Bioactive compounds from algae, such as polysaccharides, pigments, polyphenols, vitamins, lipids, and fatty acids [35], were electron donors for Ag^+^ ions. In this way, silver atoms self-organised and formed larger clusters by the bottom-up synthesis method. In this work, ground algal mass was used due to the previously proven toxic effect of nanoparticles on *C. vulgaris* metabolism [36]. The formation of AgNPs was confirmed by measuring the absorbance of nanoparticles in the visible and ultraviolet range, which showed a characteristic peak at 430 nm (absent for silver nitrate), as previously reported by Hawar et al. [34] at 435 nm for spherical nanoparticles. The spherical shape of the obtained nanoparticles is characteristic of stable AgNPs, which, in rare cases, when chemically controlled, adopt other shapes, mainly with crystalline or geometrical polygonal morphology [37,38]. A large discrepancy in the sizes of the synthesised nanoparticles is a typical phenomenon for green synthesis of nanoparticles when using *C. vulgaris* [39] and many other plants, including *Aloe vera*, *Ginkgo biloba* [40], and *Alhagi graecorum* [34]. Polydispersity plays a key role in cytotoxicity, especially the population of particles of several nanometres in the total pool of particles. According to Park et al. [41], silver nanoparticles with dimensions of 20, 80, and 110 nm and similar surface charges have different kinetics, distribution patterns, and cellular uptake; release different amounts of silver ions from their surface; and generate different amounts of ROS. Small silver nanoparticles (≤20 nm) are the most cytotoxic to both animal cells [41] and bacteria [42]. However, it is not only the size that influences toxicity but also the surface charge and surface chemistry that determine the amount of released silver ions [41]. In our previous studies, we proved that silver nanoparticles formed from *Mentha piperita* extract had smaller diameters (5–50 nm) and were more monodisperse than nanoparticles formed from *Brassica oleracea* (10–150 nm) due to the higher pH and lower temperature of the mint extract [42]. According to the literature, pH influences the reactivity of plant extract with silver ions and charge of nanoparticles [43]. High zeta potential causes the repulsion of particles in the colloid and consequently prevents the agglomeration of AgNPs [17]. The zeta potential of AgNPs apparently depends on the source of the reductant for the synthesis of nanoparticles, e.g., −19 mV for *Streptomyces* sp. [44], −18.5 mV for *Bacillus* sp. [45], and −17 mV for *C. vulgaris* [46]. Other reaction conditions are also important, such as the applied temperature or microwave radiation [46]. In our study, 30 °C for 15 min was used for the synthesis of AgNPs, and the resulting nanoparticles had a zeta potential of −15 mV, similar to the AgNPs obtained from microalgae but using microwave radiation (−17 mV). In the case of microwave energy, nanoparticles can be formed in 10 min [46], which is much shorter than our 3 h incubation period. High temperatures destabilise thermolabile enzymes involved in Ag^+^ reduction, e.g., ascorbic acid oxidase [42]. The nanoparticle colloid was purified after synthesis by 0.2 µm syringe filtration. The sample was dominated by silver (87.2%), but there were also impurities from the algae, such as oxygen (8.0%) and carbon (4.8%). According to Torabfam et al., the C–O–C, COOH, and C=O groups of *C. vulgaris* polysaccharides and proteins play the main role in the formation of functional nanoparticles [46].

In the second set of experiments, the antibacterial activity of AgNPs was tested against the most dominant bacterium in chickens—*S. enterica* serotype Enteritidis. This bacterium causes enteritis and gastritis, diarrhoea, and sepsis in consumers of eggs and poultry. The formation of biofilms in a wide temperature range and an environment with high nutrient availability poses a risk in food processing [47]. Antibacterial activity against *S. enterica* was assessed using the MBC, MIC, and PrestoBlue methods. Our studies clearly showed that AgNPs caused almost complete elimination of *S. enterica* at both low (6.75 mg/L) and high concentrations (up to 108 mg/L) after 24 h. De Emery et al. [47] reported that the antibacterial activity of AgNPs increased over time in contrast to disinfectants. Other AgNPs synthesised by chemical reduction with sodium borohydride showed bactericidal and bacteriostatic activity against *S. enterica* serotype Enteritidis isolated from duck egg faeces only at a concentration of 16 mg/L [48]. This proves that AgNPs synthesised with ecological methods are not inferior to those produced by using physical or chemical methods. In another study, 22 nm AgNPs synthesised by fungal extracts from *Phanerochaete chrysosporium* showed bactericidal activity against *S. enterica* serotype Typhimurium at a concentration of 16 mg/L [49]. It therefore seems that not only the size of the particles plays a key role but also the chemical composition of the colloid determined by the extract used.

Many mechanisms of the cytotoxic effect of silver against bacteria have been proposed in the literature, such as adhesion to cell membranes and increasing their permeability, modification of the lipid bilayer, penetration of AgNPs into the interior of cells, generation of reactive oxygen species, damage to respiratory chain, ribosomes, and vacuoles, damage to DNA, lipids, and proteins, and regulation of programmed cell death pathways [49]. The released silver ions (cations) interact with polysaccharides and lipids via electrostatic interactions due to the negative charge of the bacterial surface [50]. Such interaction leads to disruption of the membrane potential (membrane depolarisation), change in its permeability, and cytoplasmic leakage. In this way, they change the fluidity of membrane lipids. Silver nanoparticles attach and accumulate in the cell wall of Gram-negative bacteria via interactions with the components of the outer cell membrane—lipopolysaccharide and phospholipid (PE). The accumulated nanoparticles form pores in the cell wall, leading to cell death [51]. Small AgNPs are the size of the pores of the bacterial cell membrane through which they can penetrate. In addition, the nanosilver surface generates hydrogen peroxide, singlet oxygen, hydroxyl radical, and superoxide radical from the nanoparticle surface and thus damages DNA. DNA damage can be a source of mutations or cause cell death [52]. Inside the bacterial cell, the released silver ions inhibit the electron flow between cytochrome α2 and cytochrome b in the respiratory chain, thus disrupting cellular respiration. Silver also inhibits the expression of ribosomal subunits, which prevents the translation of proteins involved in adenosine triphosphate (ATP) biosynthesis, alters the functioning of respiratory enzymes associated with the cell membrane, and inhibits the activity of thiol-containing enzymes such as nicotinamide adenine dinucleotide dehydrogenase II (NADH dehydrogenase II) [51]. According to Saber et al. [53], the mechanism of silver action on microorganisms is the inactivation of bacterial enzymes, disruption of the respiratory chain, and inhibition of DNA replication. Seong et al. [54] proved that AgNPs disrupt the inner membrane of *S. enterica* by inducing the accumulation of reactive oxygen species and intracellular calcium ions. The toxicity of silver results from its oxidation and release in the form of ions in biological systems [55]. These pilot studies show that AgNPs can be used in the future to disinfect polyethylene transport crates or cages and rooms on poultry farms [47]. Moreover, the mechanisms of AgNPs are so complex that the risk of acquiring resistance is low, contrary to antibiotics [56]. The literature reports that AgNPs show stronger antibiofilm properties than unstable, short-acting disinfectants with poor bacterial penetration capacity [47].

The mechanism of silver toxicity in embryonic development is based on three pillars: (1) oxidative and nitrosative stress, (2) impaired homeostasis of the endoplasmic reticulum (ER), and (3) mitochondrial damage. Gao et al. [57] investigated the effect of silver nanoparticles on mouse embryonic stem cells. In their study, they showed that nanosilver enters the cell by endocytosis and releases silver ions from the phagosome, which generate reactive oxygen species. The continuous increase in silver ions generates hydroxyl radicals and glutathione consumption. ROS reacts with nitric oxide and forms the toxic ion ONOO^−^ and hydroxyl radical. Disturbed ROS homeostasis leads to DNA damage and causes cell cycle arrest, then if the DNA is not repaired, the cell dies an apoptotic death. Silver exposure upregulates the expression of heat shock protein and metallothionein, which is associated with the stress response and maintenance of proper protein folding [57]. Silver causes the accumulation of unfolded and misfolded proteins in the ER lumen by deregulating the proteins’ iron-response element 1, protein kinase R-like ER kinase, and activated transcription factor 6 [58]. Silver treatment increases hepatic malondialdehyde, nitric oxide, and nitric oxide synthase, causes glutathione depletion, and triggers apoptosis by increasing the expression of Bax and caspase-3 while decreasing the expression of the antiapoptotic factor Bcl2. Nitric oxide synthase increases the inflammation process in the liver via the transforming growth factor β-1 and alpha-smooth muscle actin pathway [59]. Silver also binds to the sulfhydryl groups of glutathione, thioredoxin, thioredoxin peroxidase, and superoxide dismutase, which changes the functional protein level. Mitochondrial damage by silver results from easy penetration through the inner mitochondrial membrane and damaging the dorsal structure of mitochondria. Nanosilver affects mitochondrial fission/fusion by dephosphorylating Dynamin-related protein 1 and reducing the expression of mitofusin 1 and Peroxisome proliferator-activated receptor-γ coactivator-1α. In this way, it inhibits the electron transport chain and reduces ATP production in the cell [58].

The third set of experiments aimed to investigate the potential toxicity of AgNPs in the chicken embryo model, if they were to be used for disinfection against *S. enterica*. In pilot studies, it was decided to use a lower concentration (2 mg/L) than that shown in the previous set of experiments (6.75 mg/L) due to the greater sensitivity to AgNPs at the stage of embryogenesis [60] compared to adult chickens [61]. It is also worth considering that nanoparticles would only partially enter the chickens via the dermal, oral, or inhalation route [62]. In a study by Saleh et al. [61], broiler chickens fed a basic diet supplemented with 50 ppm/kg AgNPs showed increased body weight, muscle mass, and ash digestibility and better feed efficiency. In other studies, nanosilver obtained from the alga *Jania rubens* was injected at the end of embryogenesis (day 18) at a concentration of 170 mg/L. Interestingly, despite the high concentration of AgNPs, the authors observed an improvement in the health of one-day-old chickens [63]. There is no information in the literature on the effect of the time of exposure to AgNPs from green synthesis.

Our study demonstrated that exposure to AgNPs did not significantly impact the mortality rate of 3 d old chicken embryos, which suggests a degree of biocompatibility during the initial stages of embryogenesis. No effects on mortality were also observed by Sawosz et al. [64] and Pineda et al. [65]. However, it is crucial to note that, while mortality was not affected, there was a noticeable decrease in the number of somites and angiogenesis within the chorioallantoic membrane, indicating that AgNPs could influence early developmental processes, even if they do not directly cause embryo death. Somites are formed in the process of somitogenesis in vertebrates and form from head to tail. Myotomes, scleromas, and dermatomes arise from segmental mesodermal structures, called somites. Therefore, a small difference in the number of somites may affect the pathology in the development of vertebrae, intervertebral discs, skin, and muscles in chicken embryos [66]. According to Kim et al. [67], AgNPs at a concentration of 5 mg/L caused a reduction in the number of somites in the rat embryo reared outside the mother, and this effect was dose-dependent. Similar results were obtained by Xia et al. [68] for zebrafish, in which they showed the effect of a silver dose of 5 mg/L and above on embryo deformations, mortality, and delayed hatching. In our study, AgNPs at a concentration of 2 mg/L caused embryo reduction and inhibition of normal dorsoventral flexion processes, as reported by Wang et al. [69] for nanoplastic. The antiangiogenic effect of 16 nm nanosilver obtained from sage extract was also observed by Baharara et al. [70] in a 12 d chicken embryo model. As the exposure duration increased to 16 d, the impact of AgNPs became more pronounced.

We showed that the weight of live embryos exposed to AgNPs for 16 d did not differ from the control group. Sawosz et al. [71] also demonstrated a lack of change in the mass of 20 d old embryos after administration of AgNPs at a concentration of 50 mg/L. Ognik et al. [30] documented no impact of 22 nm AgNPs administered orally at a daily dose of 5 mg/kg body weight on the mass of broilers. In other in vivo studies, the addition of AgNPs to water at doses of 10 and 20 mg/kg body weight of broilers, administered daily from day 7 to day 36 of life, did not cause changes in the mass of chickens, as shown by Pineda et al. [65]. However, after 16 d, the mortality rate of embryos increased significantly, and there were substantial changes in the weight of critical organs such as the liver and brain. Specifically, liver mass increased, while brain mass decreased, suggesting that AgNPs might induce organ-specific toxicity. This selective toxicity could be due to the differential accumulation of nanoparticles in various tissues, leading to localised oxidative stress and subsequent damage. According to Espinosa-Cristobal et al. [72], AgNPs administered orally accumulate in the small intestine, kidneys, liver, spleen, lungs, and brain, but the liver is the main organ of distribution. Therefore, the heart, apart from blood flow, does not accumulate AgNPs, and this could be the reason for unchanged mass of this organ that we observed. AgNPs, regardless of the route of administration, are taken up by macrophages and lymphatic uptake. Then, AgNPs undergo transformations in the body, i.e., they react with glutathione, sulphur, and selenium and are partially excreted in the faeces and urine [11]. Zhang et al. [28] also demonstrated an increased embryo mortality rate of 12.5% at stage 44 of development (day 18 of incubation) according to Hamburger–Hamilton guidelines [33], due to the administration of silver nanoclusters at a concentration of 3 mg/L in ovo. However, in this study, the embryos were exposed to silver from day 7 of egg incubation, which may influence higher tolerance of the embryos to the nanoparticles. The AgNPs administered in the study had a diameter of 82.2 nm, very similar to the diameter of our AgNPs (approximately 70 nm according to transmission electron microscopy, 90 nm according to the dynamic light scattering (DLS) method). The tendency of AgNPs at a concentration of 10 mg/L to increase the liver-to-body weight ratio in 18 d old embryos after in ovo administration on days 5, 11, and 17 of development was also demonstrated by Grodzik and Sawosz [73]. Similar to this study, cases of hepatobiliary system damage were observed by Recordati et al. [74]. In their research, chemically synthesised and citrate-stabilised AgNPs administered intravenously at a concentration of 10 mg/kg body weight induced size-dependent pathological effects in male CD-1 (ICR) mice (*Mus musculus*). Nanoparticles with a diameter of 10 nm caused numerous multifocal peribiliary microhaemorrhages, damage to the portal vein, and diffuse haemorrhages into the lumen of the gallbladder, whereas nanoparticles with diameters of 40 nm and 100 nm caused milder changes in the gallbladder, with congestion and wall oedema. Dosoky et al. [75] also showed pathological changes in the liver of chickens after the administration of AgNPs with silica at a dose of 8 mg, which caused mononuclear inflammatory infiltrates in the liver and kidneys, mild necrotic changes, and depletion of lymphoid cells in the spleen and thymus [75].

Damaged liver tissue releases AST, ALT, and GGT into the blood. Therefore, liver enzymes are good indicators of liver damage, inflammation, oxidative stress, apoptosis and hepatocyte damage, and impaired liver function [76]. Thus, it was necessary to determine the levels of these enzymes as a final confirmation or denial of silver toxicity. Biochemical analysis showed a significant increase in ALT levels but a decrease in AST and GGT levels in the treated group. According to norms, normal levels for chickens are 90–226 U/I for AST, 9–14 U/I for ALT, and 9–21 U/I for GGT [77,78]. Therefore, the control group was within the reference range, and silver caused subtle differences in liver parameters. Our results showed a slight increase in ALT levels in plasma, which may indicate increased hepatocyte membrane permeability. However, the levels of GGT and AST decreased slightly in the silver-treated group. Similar results were obtained by Elkloub et al. [79], who showed that a diet with the addition of 2 ppm/kg for 7 days did not affect the level of ALT and albumins and caused a decrease in AST in the blood of broilers. Also, a diet that added 0.75 mg AgNP/kg of feed in rabbits reduced the levels of AST and ALT [80]. Interestingly, in our work, no changes were observed in the level of albumins in the metabolism in which ALT is involved [81]. The levels of AST and GGT decreased compared to the references and control, which indicated the inclusion of hepatoprotective mechanisms by silver [82]. GGT as a bile duct enzyme catalysing the transfer of gamma-glutamyl glutathione to various peptide acceptors is associated with oxidative stress. Silver reduced the level of GGT—a glutathione-degrading enzyme, which could be an anti-ROS mechanism [82].

The task of antioxidants is to scavenge free radicals and chelate silver ions. The reduction in plasma antioxidant levels observed in 16 d old embryos exposed to AgNPs is a critical finding. This reduction suggests that AgNPs induce oxidative stress, which is a well-documented mechanism of nanoparticle toxicity. A significant reduction in the antioxidant capacity of blood when AgNPs were administered orally to hatched chicks was also demonstrated by Ognik et al. [30]. Similar results were obtained in other animal species, such as rabbits (*O. cuniculus*) [31] and rats (*R. norvegicus*) [32]. Liver damage is associated with a decrease in key serum antioxidant markers such as glutathione (GSH), GSH peroxidase, superoxide dismutase, and catalase [32]. GSH depletion due to nanoparticle exposure is also associated with direct AgNP-GSH coupling [83]. Another possible mechanism is phagocytosis of AgNPs, which stimulates inflammatory signalling and subsequent ROS generation in the cell [84]. Oxidative stress can damage cellular components, including lipids, proteins, and DNA, potentially leading to cell death and tissue damage. According to the literature, the effect of ROS production by AgNPs was mitigated by the addition of ascorbic acid, vitamin E, N-acetyl-L-cysteine, Trolox, gallic acid [84], selenium, chitosan [83], and many others.

Haemocompatibility studies have shown that the administration of AgNPs to chicken embryo protein has a harmful effect on red blood cells. Our observed changes in blood morphology, including polychromasia and erythrocyte membrane deformities, further support the hypothesis that AgNPs disrupt normal cellular function and integrity through oxidative mechanisms. Platinum nanoparticles, which caused the formation of knizocytes, act similarly. Knizocytes, which are immature erythrocytes with concavities in the cell membrane, are evidence that metal nanoparticles impair microcirculation and the ability to transport oxygen and carbon dioxide [85]. Only intact erythrocytes can sense the tissue demand for oxygen and release vasodilators that increase blood flow in hypoxic tissues [86]. In addition, the mechanism of oxygen transport is based on intracellular haemoglobin. Plasma haemoglobin captures nitric oxide, and causes platelet aggregation, vasoconstriction, and inflammation. After haemoglobin is released from red blood cells, haemoglobin is oxidised, and haeme dissociates into ferrihaemoglobin. Iron is then released from haeme, which causes lipid peroxidation and increases ROS [87,88]. The shape of red blood cells is also essential, and their shape deformability facilitates passage through narrow endothelial gaps. Abnormal or old erythrocytes are destroyed by macrophages [89]. In our study, ROS generation by AgNPs could also affect the oxidation of divalent iron (Fe^2+^) to trivalent iron (Fe^3+^), converting haemoglobin into methaemoglobin, which cannot bind oxygen [89]. The toxicity of AgNPs is associated with a high level of adsorption and uptake by red blood cells [90]. Adsorption of nanoparticles depends on their size and the amount of released ions, e.g., AgNPs with a diameter of 50 nm are the most toxic in the erythrocytes of fish [90]. The second key factor is the route of administration and the research model. AgNPs administered to quail at concentrations of 4, 8, and 12 mg/L via drinking water for 30 weeks did not change the amount of haemoglobin and red blood cells [91]. In another study, a decrease in haemoglobin was noted after feeding broiler chickens with silver-doped silica nanoparticles at three dietary levels (8, 16, and 20 mg/kg diet) for 35 d [92]. Chi et al. [93] reported that ionic silver is more toxic to amino acid residues than the nano form. However, AgNPs are more toxic to the secondary structure of haemoglobin (loss of alpha-helix) than ionic silver. The loss of the α-helix of haemoglobin is associated with the loss of its function in gas transport [93]. Nanosilver disrupts the functioning of erythrocytes by reducing the activity of ATPases, which consequently disrupts cellular energy metabolism, ion transport, and signal transduction [93]. The effect of silver on erythrocytes is also associated with the intercalation of silver with lipid monolayers of cell membranes and with the disruption of the secondary structure of haemoglobin, damage to membrane receptors, molecular channels, and changes in osmotic pressure [93]. Silver causes hypochromic microcytic anaemia, i.e., a reduction in the total pool of erythrocytes [59].

The haemolysis test conducted in vitro provided additional insights into the biocompatibility of AgNPs with erythrocytes. The absence of significant haemolysis after 90 min of exposure suggests that at the tested concentration, AgNPs do not cause immediate damage to red blood cell membranes. However, the in vivo observations of blood morphology changes indicate that prolonged exposure might lead to more subtle and chronic effects that are not immediately apparent in short-term assays. In the study by Chen et al. [90], AgNPs at concentrations below 20 mg/L, with diameters similar to those in our work (between 50 and 100 nm), and synthesised chemically using sodium citrate and silver nitrate, did not cause statistically significant haemolysis of erythrocytes. According to Fareed et al. [94], AgNPs with a diameter of approximately 20 nm, obtained by green synthesis and at a concentration of 7 mg/L, did not exhibit haemolytic properties. Thus, AgNPs, regardless of their synthesis method, demonstrate high biocompatibility with erythrocytes in vitro after short incubation.

## 4. Materials and Methods

### 4.1. Green Synthesis of AgNPs from Silver Nitrate and C. vulgaris

The AgNPs were synthesised by mixing 1 g of dry *C. vulgaris* biomass with 20 mL of sterile distilled water. *Chlorella vulgaris* powder was obtained from Kol-Pol (Dębica, Poland). The extract was heated in a water bath WNB7 (Memmert GmbH, Schwabach, Germany) at 95 °C for 15 min and then filtered through Whatman paper Filtrak 389 (Boeco, Hamburg, Germany). The filtered extract was centrifuged at 1200 rpm for 5 min with a Sorvall ST 16R Centrifuge (Thermo Fisher Scientific, Waltham, MA, USA), and 5 mL of the supernatant was mixed with 45 mL of a 1 mM silver nitrate solution (Chempur, Piekary Śląskie, Poland). The resulting mixture was incubated at room temperature in the dark for 3 h to produce a stable colloid of AgNPs. The colloid of nanoparticles was purified by filtration through a PES Syringe Filter with pore size of 0.22 µm (Genoplast Biotech, Rokocin, Poland). This final step eliminated organic contaminants from *C. vulgaris*. The nanoparticles were sonicated in a SONICS VC50 Sonicator (Sonics & Materials, Danbury, CT, USA) for at least 15 min before use.

#### Characterisation of AgNPs

The size, polydispersity, and zeta potential of the AgNPs were measured by using a Nano-ZS90 Zetasizer (Malvern Instruments, Malvern, Worcestershire, UK). The DLS method was used to measure particle diameter. The electrophoretic light scattering method was used to measure the charge on the particle surface. The measurements were performed in triplicate by using a AgNP colloid with a concentration of 2 mg/L.

The size, shape, and agglomeration of the AgNPs were recorded with a JEM-1220 transmission electron microscope (JEOL, Tokyo, Japan) connected to a camera (SIS Morada 11 megapixels) and operated by ITEM Olympus Soft Imaging Platform. The purified nanoparticle colloid was dropped at a concentration of 50 mg/L and a volume of 5 µL onto Formvar/Carbon 200 Mesh, Copper (Co. 201208, Agar Scientific, Essex, UK) and left to dry for 24 h. The nanoparticle diameter measurements from the microscopic images were performed using ImageJ software, version 1.54d (National Institutes of Health, Bethesda, MD, USA).

A Tecan Infinite 200 (Tecan, Durham, NC, USA) was utilised to assess the maximum absorbance of the AgNP colloid as well as silver nitrate (AgNO_3_). Absorbance measurements were conducted across the ultraviolet and visible light spectrum, ranging from 230 to 990 nm. Surface plasmon resonance of AgNPs and AgNO₃ was measured using NanoDrop™ One/OneC Microvolume UV–Vis (Thermo Fisher Scientific, Waltham, MA, USA).

The chemical composition of the AgNP colloid was assessed using scanning electron microscopy with a focused ion beam (SEM-FIB, ZEISS GeminiSEM, Ultra Plus, Germany). SEM-FIB utilised an electron and ion beam with simultaneous imaging and etching of the sample. Samples at the concentration 100 mg/L were recorded at 18 different locations in triplicate.

### 4.2. Bacterial Model

#### 4.2.1. Bacterial Culture

*Salmonella enterica* subsp. *enteritidis* (American Type Culture Collection 13076) was obtained from LGC Standard (Teddington, UK) and cultured in tryptic soy agar. Before its use in experiments, the bacterium was prepared in distilled buffer saline to the appropriate value on the McFarland scale.

#### 4.2.2. MIC and MBC Tests

The MIC test was prepared with the use of Mueller–Hinton broth in a 24-well plate. Next, 1 mL of broth was added to each well, and the hydrocolloid of AgNPs and algae (separately) were then added to the first well. Thereafter, serial dilutions were prepared. Then, 10 µL of the bacterial suspension (McFarland scale = 0.5) was added to each well. The positive control was medium with bacteria without the test factors. The MIC was evaluated on the basis of bacterial growth.

The MBC was prepared on Mueller–Hinton agar. From each well in the MIC test, the bacterial suspension was spread onto agar medium with an inoculation loop. Evaluations were made on the basis of microbial growth.

#### 4.2.3. Viability Assay

To examine the viability of bacteria under the influence of AgNPs, the PrestoBlue test (Invitrogen™, Waltham, MA, USA) was conducted. The wells in a 96-well plate were filled with 100 µL of Mueller–Hinton medium supplemented with AgNPs and algae (separately), adjusting their concentrations to 108.00, 54.00, 27.00, 13.50, 6.75, 3.38, 1.69, 0.84, 0.42, and 0.21 mg/L. Then, 10 µL of bacterial suspension (McFarland scale = 0.5) was added to each well. The control wells were medium with bacteria only. After 24 h of incubation at (37 °C), the PrestoBlue reagent (10 µL) was added to each well. The prepared plate was incubated for 30 min at 37 °C in darkness. The fluorescence was then measured with an excitation wavelength of 560 nm and an emission of 590 nm. Each well was prepared in triplicate. Results are shown as percentage viability relative to the positive control.

### 4.3. Embryo Model

#### 4.3.1. Embryo Maintenance and AgNP Injection

Fertilised chicken eggs (n = 20) from Ross Line 308 (*Gallus gallus*) were purchased from a specialised farm (Dębówka, Poland). Before the experiment, the eggs were stored at 12 °C for preservation. Eggs were then acclimatised for 12 h at room temperature immediately prior to the experiment and divided into four groups (n = five eggs per twice replicated control and AgNP treatment groups). The eggs were disinfected by washing in a supersaturated solution of potassium permanganate (Avantor Performance Materials, Gliwice, Poland) and incubating in a UVC-J1 UV Egg Sterilizer (UV KOR, Warsaw, Poland). Next, 200 µL of AgNPs at a concentration of 2 mg/L and 200 µL of phosphate-buffered saline (PBS, Sigma-Aldrich, Steinheim, Germany) were injected into the chicken egg albumen just before the beginning of incubation. Eggs were injected at two-thirds the height from the blunt end by using a tuberculin sterile syringe under the sterile conditions of a Topsafe 1.2 Laminar Flow Hood (Bioair Instruments, Siziano, Italy). Eggs receiving PBS only constituted the control group. The injected eggs were incubated for three (10 eggs, Experiment 1) or 16 d (10 eggs, Experiment 2) in a Midi 4LUX Incubator (Fest F.U.H. Waleński, Gostyń, Poland) at standard settings, i.e., 37 °C with 70% humidity and turned once per h. The two experiments served as screening studies for further application; therefore, only five eggs per study group were used.

#### 4.3.2. Experiment 1: 3 D Old Embryos

On day 3, the 10 eggs were opened. The embryos were removed by applying Whatman paper discs to them, after which the embryos were dissected together with the chorioallantoic membrane. Embryos were mounted on a glass slide and visualised using a SZX10 stereomicroscope with a camera and CellD version 3.1. software (Olympus, Hachioji-shi, Japan). The effects of AgNPs on 3 d old embryos were evaluated using the Hamburger–Hamilton classification to count somites [33]. Moreover, the ratio of live to dead or malformed embryos was recorded. ImageJ version 1.54d (National Institutes of Health, Bethesda, MD, USA) and GIMP version 2.10.22 were used to enhance somite visibility.

#### 4.3.3. Experiment 2: 16 D Old Embryos

On day 16, the 10 eggs were opened and the embryos decapitated. The number of dead or malformed embryos was recorded. The weight of the embryos and their organs were measured.

After decapitation, about 2 mL of whole blood was collected from the carotid artery of each chick and subsequently stored at 4 °C in tubes containing heparin and ethylenediaminetetraacetic acid. The blood was used to measure alanine aminotransferase, aspartate aminotransferase, gamma-glutamyltransferase, albumin, total antioxidant capacity (TAC), and in the blood smear and haemolysis tests.

##### Biochemical Analysis of Plasma

Whole blood collected from five control group embryos and five embryos treated with AgNPs was used to measure biochemical parameters in the plasma. The plasma was prepared by transferring 0.4 mL of blood from each specimen into a tube and centrifuging the tubes at 1600 rpm for 5 min. Aspartate aminotransferase, alanine aminotransferase, gamma-glutamyltransferase, and albumins were detected in serum using a Fuji Dri-Chem NX500i (Fujifilm Corporation^®^, Tokyo, Japan) according to the manufacturer’s protocol. Assays were made in triplicate using approximately 200 µL of plasma per research group.

##### TAC Assay

Whole blood collected from five control group embryos and five embryos treated with AgNPs was used to measure the TAC in the plasma. The plasma was prepared by transferring 1.5 mL of blood from each specimen into a tube and centrifuging the tubes at 1600 rpm for 5 min. Prior to TAC assay, the samples were standardised regarding protein content, using the BCA Protein Assay Kit (No. 23225, Thermo Fisher Scientific, Waltham, MA, USA). The antioxidant potential of the embryo plasma was measured using the OxiSelect™ Total Antioxidant Capacity Assay Kit (No. STA-360, Cell Biolabs, San Diego, CA, USA). To a 96-well plate, 20 µL of the blood sample and 180 µL of 1× reaction buffer were added per well. At time 0, the initial absorbance was measured with a Tecan Infinite 200 Spectrophotometer (Tecan, Männedorf, Switzerland) at a wavelength of 490 nm. Next, 50 µL of 1× ionic reagent was added per well. After 5 min, the reaction was stopped by adding 50 µL of 1× stopping solution, and the absorbance of the samples was measured again. The results were expressed as

$$\frac{{\left({\mathrm{Ab}}_{\mathrm{initial}}-{\mathrm{Ab}}_{\mathrm{final}}\right)}_{\mathrm{AgNPs}}}{{\left({\mathrm{Ab}}_{\mathrm{initial}}-{\mathrm{Ab}}_{\mathrm{final}}\right)}_{\mathrm{control}}}\times 100\%,$$

where Ab_initial_ is the initial absorbance reading, and Ab_final_ is the absorbance reading at 5 min in the appropriate treatment (control or AgNPs).

##### Blood Smear Assay

Peripheral blood obtained from 16 d old embryos was used to assess changes in the number and appearance of blood morphotic elements. Smears were made spreading 15 µL of blood on the surface of a slide with another slide. The smears were air-dried and stained using the May–Grünwald–Giemsa method (Sigma-Aldrich, Steinheim, Germany). An inverted light microscope Leica TL-LED (Leica, Wetzlar, Germany) with a digital camera (Leica MC190 HD) and LAS V4.10 software was used to take photographs at 40× magnification.

##### Haemolysis Assay

Erythrocytes obtained in “Biochemical Analysis of Plasma” Section from the control group were used for the haemolysis test. The erythrocyte pellet was washed three times with 9 mL of sterile PBS to remove leukocytes and plasma. The effect of AgNPs at a concentration of 2 mg/L on red blood cells was examined in vitro. Triton X-100 (Sigma-Aldrich, Steinheim, Germany) was used as a positive control. PBS was used as a negative control. After 90 min of incubation, the samples were centrifuged at 1600 rpm, and the haemoglobin level in the supernatant was assessed colourimetrically using a Tecan reader at a wavelength of 405 nm with 540 nm as the reference wavelength. The results were expressed as

$$\frac{{\mathrm{Ab}}_{\mathrm{AgNPs}}}{{\mathrm{Ab}}_{\mathrm{Triton}\;X\text{-}100}}\times 100\%$$

where Ab_AgNPS_ is the absorbance in the AgNPs treatment, and Ab_Triton X-100_ is the absorbance in the positive control.

### 4.4. Statistical Analysis

Statistical analyses were conducted using GraphPad Prism software, version 9.0.0 (GraphPad Software, San Diego, CA, USA). For comparisons between the two groups, the Student’s *t*-test for independent samples was used. For comparisons among more than two groups, one-way analysis of variance (ANOVA) with post-hoc Dunnett’s test was used. The results were presented as bar charts or in tabular form as mean values with their standard deviation (SD). The levels of statistical significance are indicated by asterisks: * = *p* ≤ 0.05; ** = *p* ≤ 0.01; *** = *p* ≤ 0.001; **** = *p* ≤ 0.0001. Non-significant differences are denoted by “ns”.

## 5. Conclusions

This research concept assumed the synthesis of biocidal AgNPs with possible use in the disinfection of cages and premises in the poultry industry. The first stage of this research showed that the extract from *C. vulgaris* reduces silver nitrate to AgNPs with a diameter from 20 nm to over 90 nm. In the second stage, AgNPs produced by the green synthesis method inhibited the growth of *S. enterica* at a concentration of 6.75 mg/L. In the third stage of this research, it was proven that AgNPs at a concentration of 2 mg/L present in the egg white of the chicken embryo for three days do not affect embryonic development. However, long-term accumulation of AgNPs caused high mortality of embryos, changed the weight of the liver and brain, decreased antioxidants in the plasma, and produced deformation of erythrocytes. In the future, before the biocidal product enters the market, the effect of embryotoxicity should be compared with the impact of AgNPs on chicks and adult hens and the markers of muscle development and bioaccumulation effect should be examined. We assume that adult hens will show a higher tolerance to AgNPs than embryos.

## Figures and Tables

**Table 1:** Minimal bactericidal concentration (MBC) and minimal inhibitory concentration (MIC) of silver nanoparticles (AgNPs) and algae against *S. enterica*. + = notable bacterial growth; − = no bacterial growth; +/− = partial bacterial growth. Negative control = Mueller−Hinton broth; positive control = Mueller−Hinton broth with *S. enterica*.

Concentration [mg/L]	MBC		MIC	
	AgNPs	Algae	AgNPs	Algae
108.00	−	+	−	+/−
54.00	−	+	−	+
27.00	−	+	−	+
13.50	−	+	−	+
6.75	−	+	−	+
3.38	+	+	+	+
1.69	+	+	+	+
0.84	+	+	+	+
0.42	+	+	+	+
0.21	+	+	+	+
positive control	+	+	+	+
negative control	−	−	−	−

**Table 2:** Mass (mean g ± SD) of the whole embryo, heart, brain, and liver in 16 d old chicken embryos treated with phosphate-buffered saline (control) and embryos treated with 2 mg/L of silver nanoparticles (AgNPs). Organ weights were normalised to 100 g of embryo body weight.

Body Part	Control	AgNPs	*p*-Value
Embryo	16.92 ± 0.81	17.85 ± 1.55	>0.05
Heart	0.94 ± 0.04	1.33 ± 0.39	>0.05
Brain	3.64 ± 0.53	2.35 ± 0.56	≤0.05
Liver	2.07 ± 0.09	2.60 ± 0.32	≤0.05

## Data Availability

The data presented in this study are available on request from the corresponding author.
